# Knowledge and awareness of colorectal cancer among a predominantly Indigenous Caribbean community

**DOI:** 10.1186/s12889-022-14810-5

**Published:** 2023-02-04

**Authors:** Zachary C. Warner, Kacy Gilbert-Gard, Brandon Reid, Winnie Joseph, Deanna Kepka, Priscilla Auguste, Echo L. Warner

**Affiliations:** 1 Department of Internal Medicine, University of Arizona, Tucson, USA; 2 School of Medicine, University of Arizona, Tucson, USA; 3 Department of Family Medicine, University of Kansas Wichita, Lawrence, USA; 4 Salybia Clinic, Kalinago Territory, Dominica; 5 College of Nursing, University of Utah, Salt Lake, USA; 6 Cancer Control and Population Sciences, Huntsman Cancer Institute, Salt Lake, USA; 7 Department of Family Medicine, University of Arkansas for Health Sciences, Fayetteville, USA

**Keywords:** Colorectal cancer, Screening, Prevention, Community based participatory research

## Abstract

**Objective:**

To assess awareness levels and knowledge of colorectal cancer (CRC) and CRC screening among an Indigenous Caribbean community.

**Methods:**

A community-based participatory research project was developed to perform a needs assessment of cancer prevention and education in an Indigenous non-metropolitan community in Dominica. Purposive sampling occurred at a local health clinic. Data was collected from 58 eligible patients via a 57-item structured interview. Descriptive statistics were calculated, and demographic correlates of CRC knowledge and awareness were assessed using chi-square and Fisher Exact tests.

**Results:**

Of 58 participants, 72.4% identified as Indigenous, and 36.5% had heard of CRC. Most respondents (96.1%) believed CRC screening to be “important,” yet only 3.0% of those who were age eligible had received screening in the prior 10 years, and 12.5% knew how to get screened. More respondents with incomes over $5,000 ECD had heard of CRC (72.2%) compared to those who had not (21.7%), and those who were unsure (16.7%, *p* < 0.01). Among those with a family cancer history, 14.3% knew how to get tested for CRC, 60.0% did not, and 25.0% were unsure (*p* < 0.03).

**Conclusion:**

Despite limited familiarity with CRC screening, participants broadly believed CRC screening to be important. Health education research is needed to develop patient-centered, culturally appropriate materials about CRC screening and prevention. Future work facilitating productive community partnerships and incorporating prevailing community traditions may align cancer prevention and education initiatives with community priorities.

**Supplementary Information:**

The online version contains supplementary material available at 10.1186/s12889-022-14810-5.

## Background

Colorectal cancer (CRC) is the second leading cause of cancer-related death worldwide as well as the second most common cancer among women, and third most common cancer among men [1]. It is also the third leading cause of cancer-related death in the US, and is responsible for 9.4 and 13.9% of all cancer deaths world-wide and in the US, respectively [1, 2]. Although the current incidence rate of CRC in North America is higher than the Caribbean (2.96 vs 2.03) it is expected that the next 20 years will likely result in a disproportional increase in cancer burden in the Caribbean, largely owing to the expected changes in demographics, such as life expectancy, and lifestyle changes, such as diet [3, 4]. The Pan American Health Organization estimates an increase in CRC incidence by 60% throughout the Americas, with almost double the increase in the Caribbean compared to North America [5].

Rising incidence of CRC in the Caribbean is concerning due to the fact that many Caribbean countries have limited resources to ensure equitable access to CRC screening, treatment, and survivorship care [3]. One strategy that could help improve equitable access to CRC resources is the establishment of country or region-wide cancer registries that include all peoples. The absence of country or region-wide guidelines for CRC screening and treatment in many Caribbean countries may also contribute to increased CRC morbidity and mortality in the Caribbean compared to the rest of the Americas (mortality to incidence ratio of 0.3 in North America, compared to 0.6 in LAC) [3]. Thus, we performed a cancer knowledge and awareness study to examine the prevalence of educational need for information about preventable cancers in Dominica, a small country in the West Indies.

Limited access to cancer screening and prevention information is particularly concerning for Indigenous peoples, who are often marginalized and typically have disproportionately worse health outcomes than the general population in settings across the globe. Factors contributing to poorer health outcomes include later stage at diagnosis, limited access to timely diagnosis and treatment, and lower overall knowledge of preventative and screening practices [6]. The strong association between poverty and cancer impacts Indigenous communities, who are amongst the poorest in LAC [7, 8]. Research documenting awareness of CRC, its screening practices, and cultural influences is a critical need in this region, because limited awareness of cancer prevention and screening opportunities may exacerbate the current disparities in cancer-related morbidity and mortality among Indigenous Caribbean populations. This includes suboptimal uptake of cancer prevention measures [9]. Describing Indigenous people’s knowledge and awareness of cancer may inform patient-centered and culturally based interventions that are designed to overcome these barriers.

About 10% of the Caribbean identifies as an Indigenous race or ethnicity. However, to our knowledge, no studies have evaluated knowledge and awareness of CRC prevention and screening among Indigenous Caribbean communities [6]. Therefore, we developed a community partnership to assess knowledge and awareness of preventable cancers among an Indigenous community, the Kalinago, in Dominica, West Indies. Evaluating disparities by social grouping, such as identification with an Indigenous community, is a common approach to studying inequalities in health [10]. Dominica’s population is approximately 72,000, of which about 4,000 are members of the Kalinago who reside on 3700 acres in Northeastern Dominica. This makes it the largest settlement of indigenous people in the Caribbean [11].

Informed by a constructionist-emancipatory philosophical approach, we aimed to facilitate an inclusive opportunity for community members to share their opinions and have a voice in the development of priorities for community-based cancer prevention education. A community based participatory research (CBPR) collaboration was formed between a community public health nurse, a nursing aide, tribal leadership, medical students, and public health researchers to identify cancer-related knowledge and awareness priorities [12].

## Methods

The health belief model (HBM) provided conceptual framing for this study. The HBM is a health behavior framework which has been widely used in public health research since the 1950s to describe barriers to the acceptance of preventive health screenings among adults [13]. The HBM incorporates individual perceptions, modifying factors, and likelihood of action to explain barriers to preventive health behaviors. The HBM was used as a guide for implementation of this study, selection of study questions, and dissemination of study results.

### Study design

This study was a community-based participatory needs assessment of cancer prevention education. Three community partners collaborated for the development, design, and implementation of this study. The partnership consisted of 1) local health care staff from a community clinic, including a nurse and nursing assistant, 2) a student-led non-profit consisting of first- and second-year medical students, and 3) university research faculty and staff. The community partners met multiple times prior to the study to plan the research design, recruitment, and the study materials (i.e., consent form, survey). The survey is available as a supplemental file.

### Participant recruitment & data collection

Purposive sampling occurred through a community health care clinic, with an emphasis on including all members of the Kalinago community members who attended the clinic on two given days that the research team, which included teams of 5–6 medical student research assistants led by the principal investigators, was invited to perform recruitment. The clinic where data collection occurred is open based on staffing availability, approximately once per week. Because this is the only health care clinic in an extremely rural and remote region, clinic days are attended widely and available to all members of the community. Though purposeful sampling in community-based participatory research may limit generalizability beyond the targeted sample, in our case the Kalinago, this approach elevates the relevance of participant feedback [14]. The clinic serves a population of approximately 3,000 community members, the majority of whom are descendants and/or members of an Indigenous tribe. A cross-sectional survey was administered to eligible participants from May-December 2016. Eligible participants included all adults ages 18 and older who attended the community healthcare clinic and could speak and understand English. All individuals who attended the clinic during data collection times were approached and screened for eligibility, and all were able to speak and understand English.

The study was explained to eligible participants who were offered the opportunity to participate while they waited to be seen by a healthcare provider. Each participant was matched with a trained research staff member who was a medical student and had knowledge of CRC, CRC screening, and was trained in research ethics. Research staff were trained to explain basic definitions for two questions: *“Have you ever heard of colorectal cancer? You may have also heard this called colon, rectum, or bowel cancer.”* and *“DURING THE PAST 10 YEARS, have you had any test done for colon cancer? Tests for colon cancer include stool testing such as Fecal Occult Blood Test (FOBT/FIT), colonoscopy, and sigmoidoscopy.”*. Anatomical terms such as colon and rectum were explained upon request to improve participants ability to answer accurately. Participants who were promptly seen by a provider were able to finish the survey before leaving the clinic. There were 58 survey questions and the surveys took approximately 15–30 min to complete. The research assistants emphasized that their participation or declining participation in no way influenced their or their loved one’s receipt of health services at the present or in the future. Participants were enrolled after they completed the informed consent process with a trained research assistants. Per recommendation from the community public health nurse, no compensation was provided for participation in the survey. However, participants were invited to join a health education fair at a later date where they were provided a meal to show appreciation for their time and provide feedback. The surveys were marked using pen and paper by trained research assistants based on verbal responses from participants. Surveys were then manually entered into Excel by two research assistants. Double data entry occurred on all surveys to validate data quality.

### Sociodemographic and cancer history variables

Sociodemographic variables included sex (female, male), age (18–39 years, 40–49 years, ≥ 50 years), relationship status (married/partnered, single/divorced/widow), ethnicity (Kalinago, other), education (primary school or less, high school or more), annual household income (< $5,000 ECD / < $1872 USD, ≥ $5,000 ECD/ ≥ $1872 USD), health insurance status (insured, uninsured), birth country (Dominica, other), and language (English only, multilingual (i.e., French Creole)). Cancer history variables included having a personal history of cancer (yes, no), an immediate family member with cancer (yes, no), or knowing anyone with cancer (yes, no).

### Colorectal cancer outcome variables

Three categories of outcomes were evaluated: CRC awareness and perceived susceptibility, CRC screening awareness and knowledge, and CRC testing receipt. CRC awareness and perceived risk of CRD was assessed by asking 2 questions: if participants had heard of CRC (yes, no, don’t know), and what their likelihood of being diagnosed with CRC was (Not likely at all, not likely, somewhat likely, likely, very likely). CRC screening awareness and knowledge was assessed by asking respondents 2 questions: the importance of CRC screening (Not important at all, not important, somewhat important, important, very important), and if they knew how to get tested for CRC (Yes, No/Don’t Know). CRC testing receipt was evaluated by asking if participants have received CRC screening in the last 10 years (yes, no, don’t know). There were some missing responses, and these are noted in the tables. Questions pertaining to colorectal cancer screening were derived from the 2015 US Behavioral Risk Factor Surveillance System (BRFSS) survey [15] and have previously been applied in community surveys conducted by our team among immigrant and non-English speaking participants in the United States [16, 17].

### Statistical analysis

Descriptive statistics were calculated for sociodemographic and cancer history factors. Chi square and Fisher Exact (for cells less than *n* = 5) tests were performed to evaluate sociodemographic and cancer history correlates with CRC awareness and perceived susceptibility as well as CRC screening awareness and knowledge outcomes. We limited analyses of CRC testing receipt to participants who were aged 45 and older, based on the American Cancer Society recommendations [18]. Missing data were excluded. Descriptive and inferential statistics were performed in Stata 16.

## Results

### Sociodemographics and cancer history

Of the 58 participants, the mean age was 47.1 years (SD: 14.4). As seen in Table 1, most participants were female (74.1%), aged ≥ 50 years old (46.6%), married/partnered (52.6%), had completed primary education or less (64.9%), earned less than $5,000 ECD annually (58.5%). The majority were born in Dominica (96.6%), identified with Kalinago ethnicity (72.4%), and were multilingual (60.4%). Participants were primarily uninsured (91.2%), though a slight majority did have a primary care provider (54.6%). While most participants had not been diagnosed with cancer themselves (3.4%), 50% had a family member with cancer, and 57.1% knew someone with cancer.

**Table 1:** Demographic characteristics of participants (*N* = 58)

	N	%
Sex		
Female	43	74.1
Male	15	25.9
Age		
18–39	19	32.8
40–49	12	20.7
≥ 50	27	46.6
Relationship status^a^		
Married/partnered	30	52.6
Single/divorced/widowed	27	47.4
Education^a^		
Primary school or less	37	64.9
High School or more	20	35.1
Household Income^a^		
< $5,000 ECD	31	58.5
≥ $5,000 ECD	22	41.5
Birth Country		
Dominica	56	96.6
Other	2	3.4
Ethnicity		
Kalinago	42	72.4
Other^b^	16	27.6
Language^a^		
English only	21	39.6
Multilingual	32	60.4
Health Insurance^a^		
Insured	5	8.8
Uninsured	52	91.2
Has primary care provider		
Yes	30	54.6
No	25	45.4
Personal cancer history		
Yes	2	3.4
No	56	96.6
Family cancer history		
Yes	29	50.0
No	29	50.0
Know anyone with cancer		
Yes	32	57.1
No	24	42.9

^a ^Missing for: Relationship status *n* = 1, Education *n* = 1, Household Income *n* = 5, Health Insurance *n* = 1, Language *n* = 5, Has PCP *n* = 3, Knows Someone with Cancer *n* = 2

^b ^Other included: “Black”, “Mixed”, and “Carib”

### Colorectal cancer awareness and perceived susceptibility

In Table 2, 19 participants reported that they had heard of CRC vs. 33 who had not heard of it or did not know if they had heard of it (36.5% vs. 63.5%). There were 49 participants who perceived CRC screening was either very important, important, or somewhat important compared with two who believed it was not important or not important at all (96.1% vs. 3.9%). Only one participant had received CRC testing in the last 10 years (3.0%) and seven knew how to get tested for CRC (12.5%). There were 22 participants (52.4%) who perceived their likelihood of being diagnosed with CRC as either very likely, likely, or somewhat likely, while 20 believed it was not likely or not at all likely (47.6%).

**Table 2:** Colorectal cancer awareness, importance, knowledge of testing, and perceived susceptibility (*N* = 58)

	N	%
Heard of CRC^a^		
Yes	19	36.5
No	26	50.0
Don’t know	7	13.5
Importance of CRC screening^a^		
Very important, important, somewhat important	49	96.1
Not important, not important at all	2	3.9
Received CRC test in last 10 years^b^		
Yes	1	3.0
No	30	90.9
Don’t know	2	6.1
Know how to get tested for CRC^a^		
Yes	7	12.5
No	45	80.4
Don’t know	4	7.1
Likelihood of being diagnosed with CRC^a^		
Very likely, likely, somewhat likely	22	52.4
Not likely, not at all likely	20	47.6

^a ^Missing for: heard of CRC *n* = 6, importance of CRC testing *n* = 7, received CRC test within last 10 years *n* = 1, likelihood of being diagnosed with CRC *n* = 16, know how to get tested for CRC *n* = 2

^b ^Limited to participants ages 45 and older (*n* = 34), missing for *n* = 1

### Most common reasons for being overdue for CRC testing

**Fig. 1:**
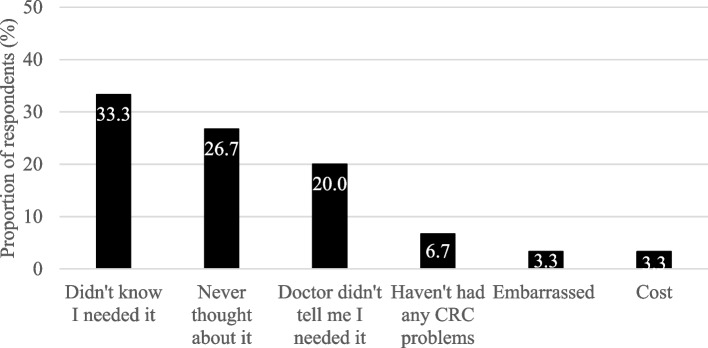
Most common reasons for being overdue for CRC testing among age eligible adults^1^ (*n* = 34). ^1^Limited to participants aged 45 and older, miss for *n* = 4

Forty-five years of age is when general U.S. guidelines specify CRC testing should begin [18]. In Fig. 1, the most common reason for being overdue for CRC testing was that participants did not know they needed CRC testing (33.3%). This was followed by having never thought about CRC testing (26.7%), and not being told by their healthcare provider they needed CRC testing (20.0%). Other reasons for being overdue for CRC testing included: they had not had any CRC problems (6.7%), feeling embarrassed (3.3%), and cost (3.3%).

### Sociodemographic correlates of CRC outcomes

In Table 3, there was a higher proportion of respondents with incomes over $5,000 ECD who had heard of CRC (72.2%) compared to those who had not (21.7%), and those who were unsure (16.7%, *p* < 0.01). A significantly higher proportion of persons who believed CRC screening was not important or not important at all were ages 40–49 years old (100%, *n* = 2 participants), compared to those who believed screening to be very important to somewhat important (16.3% *n* = 8 participants, *p* = 0.03). As shown in Table 4, Among those with a family cancer history, 14.3% knew how to get tested for CRC, 60.0% did not, and 25.0% were unsure (*p* < 0.03).

**Table 3:** Sociodemographic correlates of knowledge and awareness of colorectal cancer (*N* = 58)

	Heard of CRC *n* = 52							Importance of CRC screening^1^ *n* = 51					Received CRC test within last 10 years *n* = 34						
	Yes *n* = 19		No *n* = 26		Don’t know *n* = 7			VI,I,SI *n* = 49		NI,NAAI *n* = 2			Yes *n* = 1		No *n* = 30		Don’t know *n* = 2		
	N	%	N	%	N	%	*p*-value^3^	N	%	N	%	*p*-value^3^	N	%	N	%	N	%	*p*-value^3^
**Sex**																			
Female	15	79.0	21	80.8	4	57.1	0.48	36	73.5	2	100.0	0.55	1	100.0	20	66.7	2	100.0	0.70
Male	4	21.0	5	19.2	3	42.9		13	26.5	0	0.0		0	0.0	10	33.3	0	0.0	
**Age**																			
18–39	8	42.1	6	23.1	3	42.9	0.13	**18**	**36.7**	**0**	**0.0**	**0.03**	-	-	-	-			1.00
40–49	4	21.1	4	15.4	3	42.9		**8**	**16.3**	**2**	**100.0**		0	0.0	7	23.3	0	0.0	
≥ 50	7	36.8	16	61.5	7	36.8		**23**	**46.9**	**0**	**0.0**		1	100.0	23	76.7	2	100.0	
**Relationship status**																			
Married	9	47.4	17	65.4	3	42.9	0.43	28	57.1	1	50.0	0.68	1	100.0	18	60.0	2	100.0	0.69
Single	10	52.6	9	34.6	4	57.1		21	42.9	1	50.0		0	0.0	12	40.0	0	0.0	
**Ethnicity**																			
Kalinago	13	68.4	18	69.2	5	71.4	1.00	33	67.3	2	100.0	0.47	1	100.0	23	76.7	2	100.0	1.00
Other	6	31.6	8	30.8	2	28.6		16	32.6	0	0.0		0	0.0	7	23.3	0	0.0	
**Education**																			
≤ Primary	11	57.9	19	76.0	5	71.4	0.50	31	63.3	2	100.0	0.41	1	100.0	27	93.1	2	100.0	1.00
≥ High school	8	42.1	6	24.0	2	28.6		18	36.7	0	0.0		0	0.0	2	6.9	0	0.0	
**Household Income**																			
< $5,000 ECD^2^	**5**	**27.8**	**18**	**78.3**	**5**	**83.3**	** < 0.01**	25	55.6	1	100.0	0.56	1	100.0	19	67.9	2	100.0	1.00
≥ $5,000 ECD^2^	**13**	**72.2**	**5**	**21.7**	**1**	**16.7**		20	44.4	0	0.0		0	0.0	9	32.1	0	0.0	
**Health Insurance**																			
Insured	2	10.5	1	3.9	2	28.6	0.12	4	8.2	1	50.0	0.19	0	0.0	2	6.7	0	0.0	1.00
Uninsured	17	89.5	25	96.1	5	71.4		45	91.8	1	50.0		1	100.0	28	93.3	2	100.0	
**Birth Country**																			
Dominica	17	89.5	26	100.0	7	100.0	0.24	47	95.9	2	100.0	0.92	1	100.0	32	100.0	2	100.0	1.00
Other	2	10.5	0	0.0	0	0.0		2	4.1	0	0.0		0	0.0	0	0.0	0	0.0	
**Language**																			
English only	4	21.0	12	50.0	3	50.0	0.15	17	35.4	1	100.0	0.37	1	100.0	7	25.0	1	50.0	0.20
Multilingual	15	79.0	12	50.0	3	50.0		31	64.6	1	0.0		0	0.0	21	75.0	1	50.0	
**Has PCP**^**2**^																			
Yes	11	57.9	15	57.7	3	50.0	1.00	25	52.1	1	50.0	0.73	0	0.0	13	40.6			0.61
No	8	42.1	11	42.3	3	50.0		23	47.9	1	50.0		1	100.0	19	59.4			
**Personal cancer history**																			
Yes	0	0.0	2	7.7	0	0.0	0.63	2	4.1	0	0.0	0.92	0	0.0	2	6.7	0	0.0	1.00
No	19	100.0	24	92.3	7	100.0		47	95.9	2	100.0		1	100.0	28	93.3	2	100.0	
**Family cancer history**																			
Yes	9	47.4	17	65.4	1	14.3	0.06	27	55.1	0	0.0	0.22	0	0.0	16	53.3.0	0	0.0	0.35
No	10	52.6	9	34.6	6	85.7		22	44.9	2	100.0		1	100.0	1614	46.7	2	100.0	
**Knows someone with cancer**																			
Yes	12	66.7	13	52.0	5	71.4	0.53	29	60.4	1	50.0	0.64	0	0.0	15	53.6	2	100.0	0.34
No	6	33.3	12	48.0	2	28.615	57.7	19	39.6	1	50.0		1	100.0	13	46.4	0	0.0	

^1^ *VI* Very important, *I* Important, *SI* Somewhat important, *NI* Not important, *NIAA* Not important at all

^2^ *ECD* Eastern Caribbean Dollar, *PCP* Primary care provider

^3^ Fisher exact tests for variables with cells < *n* = 5

^4^ Missing for: Relationship status *n* = 1, Education *n* = 1, Household Income *n* = 5, Health Insurance *n* = 1, Language *n* = 5, primary care provider *n* = 3, Knows Someone with Cancer *n* = 2

**Table 4:** Demographic correlates of knowledge and awareness of colorectal cancer (*N* = 58)

	Likelihood of being diagnosed with CRC^1^ n = 42					Know how to get tested for CRC *n* = 53						
	VL, L, SL *n* = 22		NL, NAAL *n* = 20			Yes *n* = 7		No *n* = 45		Don’t know *n* = 4		
	N	%	N	%	*p*-value^3^	N	%	N	%	N	%	*p*-value^3^
**Sex**												
Female	18	81.8	15	75.0	0.43	6	85.7	33	73.3	2	50.0	0.47
Male	4	18.2	5	25.0		1	14.3	12	26.7	2	50.0	
**Age**												
18–39	8	36.4	8	40.0	0.43	1	14.3	17	37.8	1	25.0	0.49
40–49	6	27.3	2	10.0		1	14.3	10	22.2	0	0.0	
≥ 50	8	36.4	10	50.0		5	71.4	18	40.0	3	75.0	
**Relationship status**												
Married	11	50.0	11	55.0	0.75	3	42.9	24	53.3	3	75.0	0.69
Single	11	50.0	9	45.0		4	57.1	21	46.7	1	25.0	
**Ethnicity**												
Kalinago	14	63.6	15	75.0	0.43	5	71.4	32	71.1	3	75.0	1.00
Other	8	36.4	5	25.0		2	28.6	13	28.9	1	25.0	
**Education**												
≤ Primary	13	59.1	12	63.2	0.79	6	85.7	27	61.4	3	75.0	0.57
≥ High school	9	40.9	7	36.8		1	14.3	17	38.6	1	25.0	
**Household Income**												
< $5,000 ECD^2^	10	50.0	9	52.9	0.56	4	57.1	23	57.5	2	50.0	1.00
≥ $5,000 ECD^2^	10	50.0	8	47.1		3	42.9	17	42.5	2	50.0	
**Health Insurance**												
Insured	2	9.1	2	10.0	0.66	0	0.0	4	8.9	1	25.0	0.4143
Uninsured	20	90.9	18	90.0		7	100.0	41	91.1	3	75.0	
**Birth Country**												
Dominica	21	95.4	19	95.0	0.73	7	100.0	43	95.6	4	100.0	1.00
Other	1	4.6	1	5.0		0	0.0	2	4.4	0	0.0	
**Language**												
English only	10	45.4	9	56.3	0.51	1	16.7	19	45.2	0	0.0	0.14
Multilingual	12	54.6	7	43.7		5	83.3	24	54.8	4	100.0	
**Has PCP**^**2**^												
Yes	13	59.1	12	66.7	0.62	4	57.1	24	55.8	2	50.0	1.00
No	9	40.9	6	33.3		3	42.9	19	44.2	2	50.0	
**Personal cancer history**												
Yes	0	0.0	1	5.0	0.48	0	0.0	2	4.4	0	0.0	1.00
No	22	100.0	19	95.0		7	100.0	43	95.6	4	100.0	
**Family cancer history**												
Yes	8	36.4	12	60.0	0.13	**1**	**14.3**	**27**	**60.0**	**1**	**25.0**	**0.03**
No	14	63.6	8	40.0		**6**	**85.7**	**18**	**40.0**	**3**	**75.0**	
**Knows someone with cancer**												
Yes	13	61.9	13	68.4	0.67	3	50.0	27	61.4	2	50.0	0.77
No	6	33.3	14	43.7		3	50.0	17	38.6	2	50.0	

^1^ *VL* Very likely, *L* Likely, *S* Somewhat likely, *NL* Not likely, *NLAA* Not likely at all

^2^ *ECD* Eastern Caribbean Dollar, PCP Primary care provider

^3^ Chi-square test and Fisher exact tests for variables with cells < *n* = 5

## Discussion

With an expected increase in CRC in the Caribbean in the next 20 years, and probable disproportionate increase in disease burden on the Indigenous population due to social and health disparities, combating these inequities requires a basic understanding of this population’s awareness, knowledge of screening, and perceived susceptibility of CRC as well as the availability and use of prevention and screening measures [19, 20]. To our knowledge, this is the first study evaluating CRC prevention and screening among the Indigenous Kalinago community in Dominica.

Most participants in our sample had not heard of CRC and did not know how to get tested. Surprisingly, a large majority of the patients indicated that they believed in the importance of CRC testing. Although the majority of participants were over 45 years of age, only a single respondent in this age category had been tested for CRC. Although 96% of participants believed CRC screening is important, the lack of systemic national CRC screening programs in many Caribbean countries, including Dominica [20], may complicate access to CRC testing.

When asked to estimate their likelihood of CRC, 27.6% (16 respondents) declined to answer, suggesting that a sizeable portion of respondents did not feel confident in estimating their personal susceptibility to CRC. Targeted education about CRC, inherited cancer history, and CRC prevention may help improve participants’ confidence in estimating CRC susceptibility. Health education interventions may help to promote CRC screening knowledge. Once such intervention which has been growing in popularity and usage in recent years is the idea of community members as frontline health educators. These community members have a unique advantage in that they understand the social, economic, and ethnic differences of the population in a way that an outsider health professional does not. They can serve as “culture brokers,” using the trust and respect their community has of them, to more effectively disseminate health information and propagate awareness throughout their community [21]. Studies have continuously shown that community health workers in underserved communities increase positive health outcomes such as increased adherence to cancer screening guidelines, better blood pressure control, and fewer emergency department visits [22, 23]. Of even greater relevance, a 2012 study examining the effectiveness of community health care worker-delivered cancer education among a vulnerable and underserved population in Appalachian Kentucky demonstrated significantly increased knowledge of the risks and benefits of CRC, CRC screening risks and benefits, as well as an increased number of participants who had reported asking their health care provider about CRC screening [24].

There are few existing studies that have evaluated CRC awareness and knowledge in Caribbean populations, however our findings are largely consistent with at least one prior study that reported low knowledge and awareness of CRC among university students in the Caribbean [25]. Where our findings differ is that about half of the sample believed they would be diagnosed with CRC (52.4%). One possible explanation for this finding may be the belief known as fatalism, that all events are predetermined and therefore unavoidable. Fatalistic views have been documented previously among disadvantaged and marginalized populations [26]. While we did not measure fatalism in this study, this concept may in-part help explain the relatively common perception of participants that they were somewhat to very-likely to be diagnosed with CRC. Additional research is needed to determine the extent to which fatalism influences perceived susceptibility to CRC in this community.

While traditional health care workers, such as physicians and nurses, play a large role in helping to increase awareness and knowledge of cancer and value of screening tools, community health care workers can have a strong impact on health beliefs and behaviors. By virtue of being part of the community they are serving, community health workers have established trust in their community. Community health workers can effectively and broadly disseminate and reinforce important information about CRC and CRC screening in a culturally appropriate manner, which be more acceptable in Indigenous communities than other types of health behavior and health belief interventions. Community health workers can also act as liaisons between at-risk patients and the health system in order to facilitate care and screening that may not have been possible or sought out otherwise.

### Limitations

There are limitations that should be considered when interpreting our findings. Our sample was relatively small and thus findings based on small cell sizes should be interpreted with caution. The cross-sectional nature of this study precludes our ability to assess changes in knowledge and awareness of CRC or CRC testing. It is also worth noting that when we collected the data our sample was recruited from the only healthcare clinic in this community. While this means that any adult seeking care for any reason would have been offered an opportunity to participate, individuals who did not seek care from the clinic were not included and reasons for non-response were not evaluated, meaning that patterns of CRC knowledge and utilization may differ between respondents and non-respondents, including those who declined to respond to specific questions in the survey. Further data collection was abruptly interrupted due to a major natural disaster in the region (hurricane), which rendered further recruitment efforts impossible. Nonetheless, small sample research is highly valuable, despite it limits to generalizability [26]. This community engaged research was grounded in trusting relationships, prioritized the community-identified health topics of importance, and incorporated Indigenous ways of knowing and tribal customs in the design, data collection, analysis, and dissemination of results. We may also overestimate knowledge and awareness of CRC testing among this community, given that those who visited the clinic could have higher levels of engagement in healthcare services than the general population. Reporter and social desirability bias may have also influenced our results, despite efforts of the research assistants to emphasize that all answers and perspectives were valued and that no right or wrong answers existed. From a demographic perspective, our sample is overwhelmingly female; thus, our results may not accurately represent the knowledge and awareness of males in this community. Our results may not be generalizable to other Indigenous Caribbean communities given that we only recruited in a single region in Dominica, although by emphasizing purposive sampling among the Indigenous population our findings are more clearly relevant to this sub-group of the population, although less generalizable beyond this community [14]. However, as the country we recruited from is not the only LAC community impacted by CRC, our results may help other communities conduct needs assessments to identify educational gaps in CRC prevention and detection. Finally, there are limitations in using the HBM, as it does not consider the influence of the geographic setting, structural barriers, relative social power and health inequities, that potentially influence likelihood of action (i.e., likelihood of engaging in cancer prevention behaviors and testing) in Indigenous communities, which may differ from the settings in which this theory is typically applied [10].

## Conclusions

With an expected increase in CRC incidence among this population in the next 20 years, improving equitable access to cancer prevention and treatment is critical for reducing the undue burden of preventable cancer morbidity and mortality in the Caribbean. In this cross-sectional community-based participatory assessment, we found that 63.5% of participants had not heard of CRC, yet when asked, the majority of participants thought CRC screening to be important and estimated their risk of being diagnosed with CRC to be very likely, likely, or somewhat likely. Few individuals had received CRC testing in the last ten years or knew how to get screened for CRC, and while most participants had not been diagnosed with cancer themselves, half (50%) had a family member with cancer and 55.2% knew someone with cancer. Our findings suggest that community members recognize CRC screening to be important, but screening knowledge is particularly low. Only 12.5% of participants knew how to get screened for CRC. Our findings may inform the development of culturally based guidelines on improving knowledge of available CRC screenings and prevention strategies.

This study underscores an acute need for education about CRC and its screening methods in a single Indigenous Caribbean community. Descriptions of these specific barriers can inform the design and implementation of culturally based interventions addressing the anticipated disproportionate rise in CRC among the Indigenous Caribbean population. The lack of familiarity of CRC within this community is a barrier than may be overcome through the development of strong community-based health educator initiatives accompanied by the mobilization of resources from outside nations for individuals with historical disadvantage to healthcare services and health education.

## Supplementary Information

**Additional file 1:** PEAK Study.

## Data Availability

The datasets used and/or analyzed during the current study are available from the corresponding author on reasonable request.
