# Supplementary material for: Knowledge and awareness of colorectal cancer among a predominantly Indigenous Caribbean community
Source: BMC Public Health. 2023 Feb 4;23:243. doi: 10.1186/s12889-022-14810-5 (PMC9898893; doi:10.1186/s12889-022-14810-5)
Supplement: Supplementary file 1 — Additional file 1: PEAK Study. [file 12889_2022_14810_MOESM1_ESM.docx]

**PEAK Study**

Participant ID: _________________ Date: ______________

Participant ID: Date:_______/_______/______

1. **What is your age?____________________**
2. **What is your sex?**

- Male
- Female

1. **What do you do for work?**

**____________________________________**

1. **What is your marital status?**

- Married
- Living with partner
- Divorced
- Single
- Widowed

1. **What is your race or ethnicity?**

- **Kalinago**
- **Black**
- **Caucasian (White)**
- **Mixed**
- **Other:_____________________________**

1. **What country were you born in?**

**____________________________________**

1. **What country were your parents born in?**

**____________________________________**

1. **Have you ever been diagnosed with any type of cancer?**

- Yes
- No

**If yes, what type of cancer were you diagnosed with?**

**____________________________________**

1. **Has anyone in your family ever been diagnosed with cancer?**

- Yes
- No

**If yes, what type of cancer was your family member diagnosed with?**

1. **What was this family members’ relation to you?**

- Grandparent
- Parent
- Spouse
- Sibling
- Child
- Grandchild
- Aunt/Uncle
- Cousin
- Other: ___________________________

1. **Do you know anyone else who has been diagnosed with cancer?**

- Yes
- No

**If yes, how do you know them?**

___________________________

1. **How many years of schooling have you completed?**

- Primary school
- High school
- Some college
- College graduate or higher

1. **Please circle the range your total household income for one year falls.**

- Less than $5,000
- $5,000 to 9,999
- $10,000-19,999
- $20,000-29,999
- More than $30,000

1. **Do you have health insurance?**

- Yes
- No

1. **Do you have a primary health care provider (e.g. Nurse, Doctor, District Medical Officer)**

- Yes
- No

1. **If YES, have you seen your primary health care provider in the last year?**

**17. What language do you speak?**

____________________________________

- Yes
- No

**Beliefs about Cancer**

**18. How likely are you to get cancer in your lifetime?**

- Very likely
- Likely
- Somewhat likely
- Not likely
- Not likely at all

1. **How worried are you about getting cancer?**

- Very worried
- Worried
- Somewhat worried
- Not worried
- Not worried at all

1. **What do you think about this statement: “There’s not much I can do to lower my chances of getting cancer”**

- Strongly agree
- Agree
- Somewhat agree
- Disagree
- Strongly disagree

1. **Have you ever looked for information about cancer from any source?**

- Yes
- No

1. **If you have ever looked for information on cancer, where did you look for cancer information?**

- Online newspapers
- Print newspapers
- Special health or medical magazines
- Internet
- Radio
- Local Television show
- National television show
- Health Center
- Other: ______________________

**23. Do you think the following behaviors increase a person’s chance of getting cancer…?**

|  | Yes | No | Don’t know |
| --- | --- | --- | --- |
| 1. Eating fruits and vegetables |  |  |  |
| 1. Smoking |  |  |  |
| 1. Exposure to the sun |  |  |  |
| 1. Being obese |  |  |  |
| 1. Having fair skin |  |  |  |
| 1. Having many sexual partners |  |  |  |
| 1. Having a family history of cancer |  |  |  |
| 1. Being a particular race or ethnicity |  |  |  |
| 1. Pollution exposure |  |  |  |
| 1. Radon exposure |  |  |  |
| 1. Talking a lot about cancer |  |  |  |
| 1. Spending time with someone who has cancer |  |  |  |
| 1. Experiencing high amounts of stress |  |  |  |
| 1. Drinking alcoholic beverages |  |  |  |
| 1. Not exercising regularly |  |  |  |
| 1. Eating a high fat diet |  |  |  |

**Colorectal Cancer - (cancer of colon or rectum)**

**24. Have you ever heard of colorectal cancer? You may have also heard this called colon, rectum, or bowel cancer.**

- Yes
- No
- Don’t Know

1. **What do you think the likelihood is of you getting colorectal cancer?**

- Very likely
- Likely
- Somewhat likely
- Not likely
- Not likely at all

1. **Do you know how to get tested for colorectal cancer?**

- Yes
- No
- Don’t Know

1. **DURING THE PAST 10 YEARS, have you had any test done for colon cancer? *(Tests for colon cancer include stool testing such as Fecal Occult Blood Test (FOBT/FIT), colonoscopy, and sigmoidoscopy)***

- Yes
- No
- Don’t Know

**If yes, what type of test did you have?**

- Stool Test (Fecal Occult Blood Test FOBT/FIT)
- Colonoscopy
- Sigmoidoscopy
- Don’t know
- Other: ________________________

**If yes, when? _________________________________**

1. **How would you rate the importance of getting a colorectal cancer screening (colonoscopy/sigmoidoscopy or stool test such as FOBT/FIT test)?**

- Very important
- Important
- Somewhat important
- Not important
- Not important at all

1. **If you haven’t had a test for colon cancer in the last 10 years, what is the ONE most important reason why?**

- Too young
- No reason/never thought about it
- Didn’t know I needed this type of test
- Doctor didn’t tell me I needed it
- Haven’t had any problems
- Put it off
- Too expensive/no insurance/cost
- Too painful, unpleasant
- Embarrassing
- Don’t have a doctor
- Other: ___________________________
- Don’t know
- Not applicable, I have had a CRC in the last 10 years

**Other Cancer Questions**

1. **Have you heard of Human Papillomavirus or HPV? *HPV is not the same as HIV*.**

- Yes
- No
- Don’t know

1. **HPV is able to cause cervical cancer:**

- True
- False
- Don’t know

1. **Most people have HPV at some point in their lives:**

- True
- False
- Don’t know

1. **Before today, have you heard of the HPV vaccine (also known as the cervical cancer vaccine or Gardasil)?**

- Yes
- No
- Don’t know

1. **If yes, where did you hear about the HPV vaccine? (check all that apply)**

- Friends
- Family
- Healthcare professional (doctor or nurse)
- Television
- Radio
- Newspaper
- Internet
- Planned Parenthood
- Other: _________________________

1. **The HPV vaccine has 1 dose:**

- True
- False
- Don’t know

1. **How long has it been since you last visited a dentist or dental clinic?**

- **I have never gone to a dentist or dental clinic**
- **Within the past year**
- **Within the past 2 years**
- **Within the past 5 years**
- **More than 5 years ago**

1. **In general, would you say your nutrition habits are:**

- Excellent
- Very good
- Good
- Fair
- Poor

1. **How comfortable are you making an appointment, or visiting with a doctor or other health care providers?**

- Very comfortable
- Comfortable
- Somewhat comfortable
- Not comfortable
- Not comfortable at all

1. **In general, would you say your health is:**

- Excellent
- Very good
- Good
- Fair
- Poor

1. **Do you currently smoke?**

- Yes
- No

***WOMEN CONTINUE TO NEXT PAGE 🡪***

***THIS SECTION FOR MEN ONLY***

1. **Have you ever received screening for prostate cancer?**

- **Yes**
- **No**

1. **If you responded “No” above, please explain why you have not received a screening for prostate cancer?**

**THANK YOU FOR PARTICIPATING!**

**We appreciate your responses!**

**THIS SECTION FOR WOMEN ONLY**

**Breast Cancer**

1. **What do you think the likelihood is of you getting breast cancer?**

- Very likely
- Likely
- Somewhat likely
- Not likely
- Not likely at all

1. **Do you know how to get tested for breast cancer?**

- Yes
- No
- Don’t know

1. **How would you rate the importance of getting a mammogram?**

- Very important
- Important
- Somewhat important
- Not important
- Not important at all

1. **Have you had a Mammogram DURING THE PAST 2 YEARS?**

- Yes
- No
- Don’t know

1. **If you haven’t had a mammogram in the last 2 years, what is the ONE most important reason why?**

- Too young
- No reason/never thought about it
- Didn’t know I needed this type of test
- Doctor didn’t tell me I needed it
- Haven’t had any problems
- Put it off/laziness
- Too expensive/no insurance/cost
- Too painful, unpleasant
- Embarrassing
- Don’t have a doctor
- Other: ___________________________
- Don’t know
- Not applicable, I have had a mammogram in the last 2 years

**How much do you agree with the following statements?**

1. **If someone hits my breast, I will get breast cancer.**

- Strongly agree
- Agree
- Somewhat agree
- Disagree
- Strongly disagree

1. **Women who have large breasts are more likely to get breast cancer than women who have small breasts.**

- Strongly agree
- Agree
- Somewhat agree
- Disagree
- Strongly disagree

1. **Breastfeeding a baby can protect you from getting breast cancer**

- Strongly agree
- Agree
- Somewhat agree
- Disagree
- Strongly disagree

1. **Are all breast lumps cancer?**

- Yes
- No
- Don’t know

**Cervical Cancer Questions**

1. **Have you heard of cervical cancer?**

- Yes
- No
- Don’t know

1. **Do you know what a Pap smear is?**

- Yes
- No
- Don’t know

1. **How often should you get a Pap smear?**

- Only if you think you have cancer
- Every year
- Every three years
- Every five years

1. **What do you think the likelihood is of you getting cervical cancer?**

- Very likely
- Likely
- Somewhat likely
- Not likely
- Not likely at all

1. **How would you rate the importance of getting a Pap smear?**

- Very important
- Important
- Somewhat important
- Not important
- Not important at all

1. **If you haven’t had a Pap smear in the last 3 years, what is the ONE most important reason why?**

- Too young
- No reason/never thought about it
- Didn’t know I needed this type of test
- Doctor didn’t tell me I needed it
- Haven’t had any problems
- Put it off/laziness
- Too expensive/no insurance/cost
- Too painful, unpleasant
- Embarrassing
- Don’t have a doctor
- Other: ___________________________
- Don’t know
- Not applicable, I have had a Pap smear in the last 3 years

**58. Please mark True or False…**

|  | True | False | Don’t know |
| --- | --- | --- | --- |
| **You cannot get a pap smear while on your period** |  |  |  |
| **You need a pap smear even if you have not had sex** |  |  |  |
| **You do not need a pap smear if you are not sexually active** |  |  |  |

**THANK YOU FOR PARTICIPATING!**

**We appreciate your response**
